# An Automated Detection Method for Motor Vehicles Encroaching on Non-Motorized Lanes Based on Unmanned Aerial Vehicle Imagery and Civilized Behavior Monitoring

**DOI:** 10.3390/s26072027

**Published:** 2026-03-24

**Authors:** Zichan Tan, Yin Tan, Peijing Lin, Wenjie Su, Tian He, Weishen Wu

**Affiliations:** 1 School of Electronic Information, Guilin University of Electronic Technology, Beihai 536000, China; tan_zichan@mails.guet.edu.cn; 2 School of Computer Engineering, Guilin University of Electronic Technology, Beihai 536000, China; tanyin@guet.edu.cn (Y.T.); hoteam_26@163.com (T.H.); 18278960036@163.com (W.W.)

**Keywords:** UAV-view traffic monitoring, oriented object detection, lane region segmentation, spatio-temporal debouncing, false alarm suppression

## Abstract

Motor vehicle encroachment into non-motorized lanes is a common but hard-to-verify violation in urban intersections, especially when monitored from unmanned aerial vehicles (UAVs) or high-mounted overhead views. Existing rule-based solutions built on horizontal bounding boxes and center-point/line-crossing criteria are sensitive to perspective distortion, occlusion, and frame-to-frame jitter, resulting in unstable decisions and low evidential value. This paper presents a cascaded UAV-view system that closes the loop from perception to evidence output through detection–segmentation–recognition–decision. First, we adopt a two-stage detection cascade: a lightweight vehicle detector localizes vehicles using axis-aligned bounding boxes, and a dedicated YOLOv5n-based oriented bounding box (OBB) license plate detector, constructed via architecture grafting and weight transfer, is then applied within each vehicle region of interest (ROI) to localize rotated license plates under large pose variation and small-target conditions. Second, a U-Net lane region segmentation module provides pixel-level spatial constraints to define an enforceable lane occupancy region. Third, a perspective rectification step is integrated with the PP-OCRv4 optical character recognition (OCR) framework to improve license plate recognition reliability for tilted plates. Finally, an area ratio criterion and an N-frame temporal counter are used to suppress transient misdetections and stabilize alarms. On a representative 100-sample controlled encroachment benchmark, the proposed system improves detection accuracy from 67.0% to 92.0% and reduces the false positive rate from 32.35% to 5.88% compared with a baseline horizontal bounding box (HBB)-based rule. The system outputs both violation alarms and license plate evidence, supporting practical deployment for multi-view traffic governance.

## 1. Introduction

With the rapid growth of urban traffic demand and motor vehicle ownership, Intelligent Transportation Systems (ITSs) have become essential for congestion mitigation, road safety improvement, and refined traffic governance [1]. Vision-based violation detection has therefore been widely deployed for video patrol, smart law enforcement, and refined road resource management [2,3]. Among these violations, motor vehicles encroaching into non-motorized lanes (e.g., bicycle lanes) represent typical spatial–semantic behaviors: a system must detect vehicle targets, delineate the enforceable lane region, and make temporally stable decisions under complex illumination, occlusion, and viewpoint variations [4,5].

In practical governance, bicycle lane encroachment not only impedes non-motorized traffic efficiency but also increases collision risk for vulnerable road users. Such violations are frequent at intersections, bus stops, and tidal-flow zones and often appear as short-duration yet recurring events. For enforcement systems, overly strict thresholds can amplify false positives caused by brief boundary touches and increase manual review costs, whereas overly loose thresholds may miss substantive encroachments, undermining governance effectiveness. Therefore, field-deployable algorithms should prioritize both single-frame correctness and temporally stable, interpretable evidence output.

Early license plate recognition systems mainly relied on conventional image processing, including edge detection, morphological operations, and color/texture feature extraction [6,7]. These pipelines are sensitive to illumination variations and background clutter, and their robustness often degrades under uncontrolled conditions such as low illumination, worn plates, and complex backgrounds [8]. Recent advances in deep learning have enabled convolutional neural network (CNN)-based detection and recognition to replace hand-crafted methods and become the mainstream paradigm for detection and optical character recognition in traffic scenes [9].

From a task viewpoint, encroachment detection couples detection, lane/region segmentation, and temporal decision making: detection identifies vehicle targets, segmentation defines enforceable lane regions, and decision logic determines whether the behavior meets regulatory thresholds. Large-scale driving datasets and multi-task learning have improved modeling of road elements. For example, BDD100K provides multi-task annotations for detection, segmentation, and tracking, offering a foundation for learning and evaluating road-related regions [10]. However, for rule-driven applications such as lane encroachment enforcement, general semantic categories still require secondary modelling and system-level integration.

In the field of object detection, single-stage detectors like YOLO (You Only Look Once) are widely adopted for vehicle and license plate localization tasks due to their exceptional balance of real-time performance and detection accuracy [11,12]. Meanwhile, in text recognition, convolutional recurrent neural networks (CRNNs) and their variants—particularly Baidu’s PaddleOCR framework—have achieved significant results in Scene Text Recognition (STR) tasks due to their lightweight nature and high accuracy [13,14].

Although existing techniques achieve high detection accuracy on standard datasets such as CCPD and the Application-Oriented License Plate (AOLP) dataset [15], automatic identification of “motor vehicles encroaching on non-motorized lane” in real-world road scenarios still faces three key challenges:

1. Simultaneous understanding of targets and regions: Detecting vehicles alone is insufficient to determine encroachment; pixel-level characterization of the non-motorized line area is required.
2. Perspective and projection errors: Elevated/bird’s-eye view angles cause spatial misalignment between vehicle projections and lane markings, rendering simple line-crossing logic unreliable.
3. Dynamic interference and temporal stability: Tree shadows, occlusions, image jitter, and short-term false detections destabilize single-frame classification [16,17], necessitating spatio-temporal consistency constraints to reduce false positives.

Furthermore, perspective variation represents the critical gap between treating this as a “standard academic task” versus achieving “real-world engineering deployment.” Fixed ground surveillance typically employs level or slightly downward angles, where the geometric relationship between vehicles and lane markings remains relatively stable. Conversely, aerial or high-mounted overhead viewpoints capture vehicles oriented arbitrarily within the image, and the bounding boxes surrounding vehicles introduce substantial background noise. This renders geometry-based rules centered on “line-crossing/center-point detection” non-robust. Rotation detection for arbitrarily oriented objects (Oriented Object Detection) thus emerges as a critical technical pathway: one class of representative methods directly generates high-quality rotation candidates within a two-stage framework (e.g., Oriented R-CNN), enhancing localization quality and efficiency for objects in any orientation [18]; another class explicitly models rotational transformations at the feature level (e.g., ReDet) to reduce reliance on extensive rotation data augmentation and improve directional generalization in aerial scenes [19]. Collectively, these studies demonstrate that in top-down/aerial scenarios, using OBBs (oriented bounding boxes) better aligns with the true boundaries of objects compared to HBBs (horizontal bounding boxes), thereby establishing a geometric foundation for subsequent region overlap calculations and evidence extraction.

In recent years, multi-source video fusion from fixed surveillance and drone inspections has become a significant trend in traffic governance [20]. Directly transferring existing general-purpose models to such scenarios often leads to missed detections or misclassifications [21]. Therefore, constructing a visual alert system capable of adapting to multi-view conditions and producing interpretable judgment results holds practical significance.

Beyond spatial geometry, temporal consistency equally determines whether system alerts are “actionable.” In real-world footage, jittery detection boxes, flickering lighting, and brief occlusions cause single-frame judgments to oscillate frequently between 0 and 1, significantly increasing false alarm rates. In practice, common strategies involve introducing object tracking or cross-frame correlation to elevate “single-frame judgments” to “trajectory-level judgments,” using continuity constraints to filter out sporadic noise. ByteTrack in the multi-object tracking domain proposed the approach of “fully utilizing low-confidence detection boxes for correlation,” which reduces trajectory breaks and enhances correlation stability in occlusion and low-score scenarios [22]. Integrating such tracking/association mechanisms with violation rules enables more natural implementation of engineering criteria like “continuous N-frame confirmation” and “sustained encroachment duration.” This significantly reduces false positives and enhances alert credibility within acceptable latency.

To address these challenges, this study proposes a visual alert system for motor vehicle encroachment on non-motorized lane from a drone perspective. License plate detection and character recognition serve as evidence enhancement modules, cascaded with the encroachment determination process and specifically optimized for complex traffic scenarios observed from a drone viewpoint. We employ a transfer learning strategy, modifying YOLOv5′s anchor mechanism and configuration files to construct a “hybrid” model adapted for small-object and multi-angle detection, addressing insufficient generalization capabilities under specific perspectives [23]. Furthermore, considering that existing public datasets predominantly feature ground-level or eye-level perspectives with limited high-altitude bird’s-eye samples, this study constructed a proprietary drone license plate dataset encompassing diverse lighting conditions, angles, and occlusion scenarios [24]. By conducting mixed training and comparative testing with the publicly available CCPD dataset, we validated the proposed method’s superior adaptability within specific domains [25].

Novelty relative to prior work: Existing unmanned aerial vehicle (UAV) traffic monitoring studies typically focus on perception tasks (e.g., detection/tracking or behavior recognition) and report accuracy on vision benchmarks, but they often stop short of producing enforceable evidence and stable, rule-consistent decisions under overhead-view jitter and perspective bias. In contrast, we formulate UAV-view lane encroachment as an end-to-end “perception → decision → evidence” workflow and present a practical closed-loop pipeline. Our novelty lies in (i) a two-stage detection cascade tailored to UAV viewpoints (vehicle HBB for occupancy computation, followed by OBB-preferred plate localization within each vehicle region of interest (ROI) for evidence), (ii) region-based encroachment reasoning using lane region segmentation rather than center-point/line crossing, and (iii) spatio-temporal debouncing with evidence-triggered OCR to suppress transient alarms while preserving evidential reliability.

The main contributions of this paper are summarized as follows:

1. UAV-oriented enforcement dataset and protocol: We curate a cross-view dataset for non-motorized lane encroachment, consisting of ~800 high-resolution frames/images (including ~500 UAV-view samples). The data cover multiple intersections, flight heights, and tilt angles, as well as diverse lighting/occlusion conditions. We adopt a scene-wise split by video sequence/intersection to reduce temporal leakage and annotate (i) vehicle HBBs and plate OBBs and (ii) a binary enforceable lane region mask.
2. Hybrid YOLOv5n-OBB for rotated plate localization under UAV views: We develop a lightweight hybrid detector via architecture grafting and weight transfer, using a stable YOLOv5n backbone with an Anchor-Free OBB head to localize rotated license plates under large pose variation and small-target conditions. In the end-to-end pipeline, vehicles are detected with axis-aligned boxes for occupancy computation, while plate OBBs provide cleaner crops for rectification and OCR evidence.
3. Closed-loop decision with evidence output: We integrate lane region segmentation (U-Net) with an area ratio encroachment criterion and an N-frame temporal counter to suppress transient misdetections and stabilize alarms. To improve evidential value under UAV tilt, we apply perspective rectification on plate OBBs and perform evidence-triggered OCR, producing “alarm + readable plate evidence” outputs suitable for practical governance.

The structure of this paper is as follows: The Related Work section reviews and summarizes the latest advancements and limitations of deep learning-based license plate detection and recognition technologies. The Methodology section details the design of the improved YOLOv5 detection network, the PaddleOCR recognition module, and the dataset construction process. The Experiments section presents the experimental setup, evaluation metrics, comparative results, and ablation analysis. The Discussion section provides an in-depth analysis of experimental phenomena, model limitations, and potential failure cases. Finally, the Conclusion section summarizes the research findings and outlines future research directions.

## 2. Research Advances in Lane Violation Detection and License Plate Recognition Technology

This section reviews the evolution of key technologies related to motor vehicle encroachment detection systems on non-motorized lane. It focuses on analyzing the application of vehicle object detection, lane line and region segmentation techniques in traffic scenarios, as well as the current development status of optical character recognition (OCR) technology in license plate recognition. With the rapid advancement of Intelligent Transportation Systems (ITSs), automated detection of lane violations increasingly relies on computer vision and deep learning methods, particularly for lane recognition, vehicle behavior analysis, and license plate recognition in complex traffic environments. Traditional detection methods struggle to address the challenges posed by diverse scenarios and viewing angles in the specific context of motor vehicles encroaching on non-motorized lane. Consequently, new approaches must not only achieve higher detection accuracy but also demonstrate robust scene understanding capabilities and robustness.

### 2.1. Deep Learning-Based Object Detection Techniques

Object detection forms the cornerstone of Intelligent Transportation Systems. While early two-stage detectors like Faster R-CNN [26] offer accuracy advantages, their complex region proposal extraction process struggles to meet real-time monitoring performance demands. In contrast, single-stage detectors such as the Single Shot MultiBox Detector (SSD) [27] and the YOLO series models have led to substantial improvements in inference speed. In license plate recognition, traditional horizontal bounding boxes (HBBs) struggle with perspective distortion from drone perspectives. Addressing this, Oriented Object Detection (OBB) has emerged as a research focus. In recent years, the engineering community has begun integrating OBB detection capabilities into YOLO variants. For instance, Ultralytics provides OBB task configuration and training/inference workflows in YOLOv8. Concurrently, high-precision regression losses like distribution focal loss (DFL) are employed to enhance boundary localization precision [28]. Recent UAV-based traffic surveillance studies have also analyzed traffic behavior and violations from drone video streams [29], highlighting the importance of robustness under overhead views and motion jitter. In addition, survey work has summarized the unique challenges of UAV imagery for object detection (small objects, viewpoint changes, and scale variation) [30]. However, in specific industrial-grade applications, model performance depends not only on architectural sophistication but also on adaptability to particular datasets. Research indicates that “hybrid” models constructed via weight transfer (such as the improved YOLOv5n-OBB proposed herein) often exhibit superior convergence speed and mean average precision (mAP) in small-sample scenarios compared to higher-version models that suffer from undertraining due to early stopping [31].

### 2.2. Semantic Segmentation and Region Classification in Road Scenes

For detecting the specific behavior of “motor vehicles encroaching on non-motorized lanes,” existing research primarily falls into two categories: methods based on lane line detection and methods based on region segmentation. Traditional lane line extraction often relies on the Hough Transform or Canny edge detection [32], but these methods are prone to noise and missed detections when encountering tree shade, intense light fluctuations, or irregular bicycle lane markings [33]. Modern approaches like LaneNet or SCnn [34] perform better in complex environments, yet their high computational overhead limits edge device deployment. Meanwhile, multi-task models such as YOLOP [35] attempt simultaneous detection and segmentation within a single framework, though they still fall slightly short of dedicated segmentation networks in pixel-level precision. For overhead/UAV imagery, lane extraction and lane boundary segmentation have also been explored using deep learning on UAV images [36]. U-Net [37], leveraging its symmetric encoder–decoder architecture and skip connections, excels at capturing minute semantic features. It initially demonstrated exceptional few-shot learning capabilities in medical imaging with scarce samples. For targets like non-motorized lane possessing contiguous regional properties, U-Net’s pixel-level segmentation more accurately defines “violation zones” than simple line detection. Several review studies indicate that when training samples are relatively limited, relatively simple encoder–decoder networks with skip connections (such as U-Net) are often easier to train and offer better practicality [38].

### 2.3. Optical Character Recognition (OCR) and License Plate Recognition

License plate character recognition constitutes the final stage of violation evidence collection. Drone-based automatic license plate recognition has also been studied, but it remains challenging under aerial viewpoints and complex environmental conditions [39]. While traditional OCR engines like Tesseract [40] support multilingual processing, they exhibit high false positive rates when identifying plates with cluttered backgrounds, uneven lighting, or geometric distortions. The classic convolutional recurrent neural network (CRNN) plus connectionist temporal classification (CTC) framework [13], though possessing strong sequence modeling capabilities, lacks spatial flexibility when handling tilted or distorted text. In recent years, recent frameworks like PaddleOCR [41] have introduced more robust detection algorithms (e.g., DBNet) and recognition models. Compared to traditional license plate recognition (LPR) systems [21], PaddleOCR incorporates built-in image preprocessing and denoising modules that effectively correct perspective distortion caused by drone shooting angles. Furthermore, PaddleOCR’s lightweight pre-trained models significantly reduce model size while maintaining high accuracy, addressing the challenge of real-time operation on mobile surveillance devices [42].

### 2.4. Current Research Status of Traffic Violation Detection Systems

Current ITS research primarily focuses on red-light running [43] and speeding detection [44]. The former relies on temporal synchronization between vehicle detection and traffic signal states, while the latter emphasizes one-dimensional velocity vector estimation. In contrast, detecting motor vehicle encroachment into non-motorized lane involves complex scene semantic understanding, resulting in limited literature. Existing approaches predominantly employ simple “lane departure detection” logic, which judges whether a vehicle’s center crosses lane markings [4]. Similar methodologies are also applied in scenarios like illegal parking detection [45]. However, this logic fails significantly in drone bird’s-eye views, as the elevated perspective causes displacement errors between vehicle projections and lane markings. Recent research has integrated lane classification, object tracking, and violation rules into a unified framework to enhance the stability and interpretability of lane-based violation detection in real-world videos. By precisely segmenting non-vehicle lanes and calculating the Intersection over Union (IoU) between these segments and violation regions using the rotation box parameters $\left(x,\;y,\;w,\;h,\theta \right)$ from OBB models, false positives can be significantly reduced, thereby enhancing the reliability of enforcement evidence [46].

## 3. Methodology

### 3.1. System Architecture and Data Flow

#### 3.1.1. Overall System Architecture Design

**Figure 1:**
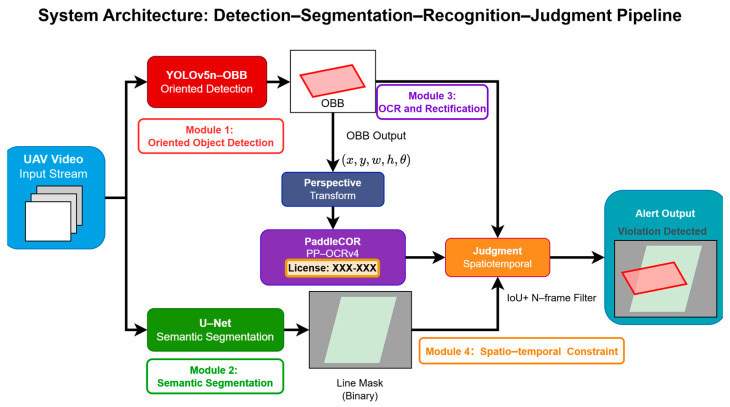
System-wide technology roadmap.

To address the demand for high-precision detection of motor vehicles illegally occupying non-motorized lanes from a drone perspective, this study proposes a cascaded visual perception framework based on “detection–segmentation–recognition–classification.” The system aims to overcome challenges in high-altitude overhead scenarios, including license plate perspective distortion, irregular lane boundaries, and dynamic interference (e.g., tree shadows and lighting variations). As illustrated in Figure 1, the system’s data processing pipeline primarily comprises the following four core submodules:

Oriented Object Detection Module: The system employs two separate detectors. A vehicle detector first localizes vehicles using axis-aligned bounding boxes (HBBs). Then, within each vehicle ROI, a dedicated license plate OBB detector (YOLOv5n-OBB) localizes rotated plates and outputs either quadrilateral corner points or $\left(x,y,w,h,\theta \right)$ parameters for subsequent rectification and OCR.

Semantic Segmentation Module: This module utilizes a fully convolutional U-Net network for pixel-level classification, generating high-precision binary masks for non-motorized vehicle lanes to address the robustness limitations of traditional edge detection algorithms.

Character Recognition and Rectification Module: This system corrects tilted license plates using geometric projection transformations and incorporates the PP-OCRv4 Server model to achieve highly reliable character sequence recognition.

Spatio-temporal Constraint Module: This module combines the area ratio criterion with a temporal counter to logically filter instantaneous detection results, outputting final violation alerts.

Figure 1 illustrates the overall architecture and data flow of the proposed system. The system takes drone video streams as input. First, a vehicle detection module localizes vehicle regions in each frame. Then, a dedicated license plate detector is applied within each vehicle ROI to localize plates (supporting OBB outputs). In parallel, the U-Net module segments the non-motorized lane region. Subsequently, the system performs character recognition (OCR) on the license plate regions to obtain license plate numbers and confidence scores. Finally, the spatio-temporal decision unit integrates information including “object detection results–lane segmentation areas–cross-frame stability/continuity constraints” to determine lane encroachment and output violation alerts with supporting evidence. This cascaded framework decouples perception, segmentation, recognition, and decision-making modules, enabling replacement or upgrading of individual components across different scenarios without disrupting the overall workflow.

#### 3.1.2. Data Flow and Network Input/Output Process

(1) Input Definition and Preprocessing

System inputs consist of video frames or static images captured by intersection surveillance cameras or drones, denoted as $I_{t}\in R^{H\times W\times 3}$. To balance spatial accuracy in detection and segmentation, this paper employs uniform resolution scaling and normalization for input frames, preserving the original scale and scaling ratio for subsequent coordinate mapping. When processing video streams, frame indices are denoted as t, and a temporal state cache for targets is maintained (comprising counters and associated key-value pairs).

(2) Two-Stage Detection: Vehicle Detection and License Plate OBB Localization

Given frame $I_{t}$, we first apply a vehicle detector to obtain axis-aligned vehicle boxes. For each detected vehicle ROI, we then apply a dedicated license plate OBB detector to localize the plate region:

(1) $$V_{t}={\left\{\left(x_{i},y_{i},w_{i},h_{i},s_{i},{id}_{i}\right)\right\}}_{i=1}^{N_{t}^{v}},$$

(2) $$P_{t}^{\left(i\right)}={\left\{\left(b_{ij},s_{ij}\right)\right\}}_{j=1}^{N_{t,i}^{p}},\;b_{ij}=\left(x_{ij},y_{ij},w_{ij},h_{ij},\theta_{ij}\right)or(x_{1},y_{1},...x_{4},y_{4})$$

where $\left(x_{i},y_{i},w_{i},h_{i}\right)$ denote the axis-aligned vehicle bounding box parameters, $s_{i}$ is the confidence score, and ${id}_{i}$ is the tracking association identifier produced by the vehicle tracker.

For the license plate detector, $b_{ij}$ denotes an oriented bounding box (OBB) of the j-th plate within the i-th vehicle ROI, represented either by $\left(x_{ij},y_{ij},w_{ij},h_{ij},\theta_{ij}\right)$ or by four vertices $\left(x_{1},y_{1},...x_{4},y_{4}\right)$, and $s_{ij}$ is the corresponding confidence score. The recognized plate evidence is associated with the corresponding vehicle track ${id}_{i}$.

The detection stage provides inputs to two subsequent branches.

(i) Intrusion detection: Vehicle boxes $V_{t}$ are used to compute the overlap ratio between each vehicle region and the segmented non-motorized lane area.

(ii) Evidence enhancement: For each detected vehicle, the license plate detector localizes plate OBBs $P_{t}^{\left(i\right)}$, which are further rectified and recognized by OCR to generate readable plate evidence.

This two-stage cascade reduces the search space for small plates and improves both efficiency and robustness under UAV viewpoints.

(3) Semantic Segmentation: Non-Motor Vehicle Lane Region Segmentation (U-Net)

Frame $I_{t}$ is input in parallel to the U-Net segmentation network, producing a binary mask for non-motorized lane:

(3) $$M_{t}\in {\left\{0,1\right\}}^{H\times W},$$

Subsequently, connected-component filtering and contour extraction are applied to $M_{t}$ to derive a polygonal representation of the non-motorized lane region, denoted as $L_{t}$ (lane polygon), which is used for subsequent spatial overlap calculations. Compared with using lane-line geometry rules alone, this pixel-level region description provides a more stable basis for defining the violation zone under tree shadows, occlusions, and worn or degraded lane boundaries.

Implementation details: The binary lane mask is converted to a polygon by extracting the outer contour with OpenCV findContours (RETR_EXTERNAL and CHAIN_APPROX_SIMPLE). If the segmentation model directly outputs polygon coordinates (e.g., result.masks.xy in Ultralytics), those polygon vertices are used directly without an additional contour extraction step. Encroachment is computed at the polygon level rather than by pixel-wise mask counting. Specifically, the lane region is represented as a polygon, and the vehicle is represented as a horizontal bounding box (HBB). The overlap area is computed using Shapely (GEOS backend) as the intersection area between the lane polygon and the vehicle box, and the encroachment coefficient is defined as the ratio of this intersection area to the vehicle box area. Both the lane polygon and the vehicle box are expressed in the original image coordinate system: although the lane segmentation model internally resizes the input, its output is restored to the original image size before contour extraction, and the vehicle detector also outputs coordinates in the original image space. Area computation therefore requires no additional coordinate remapping. The intersection area is computed as a floating-point area in Shapely, and a vehicle is flagged as encroaching when the overlap ratio exceeds the intrusion threshold (default τ=0.20). To suppress transient alarms caused by frame-level jitter near the threshold, the online pipeline further applies a consecutive-frame confirmation rule (intrusion_min_frames, default: 3 frames), without additional epsilon or hysteresis compensation.

(4) Spatial Determination: Area Ratio Criterion Calculation

For each vehicle target $B_{t}^{\left(k\right)}$, calculate its overlap area ratio (encroachment coefficient) with the non-motorized lane area $L_{t}$:

(4) $$R_{t}^{\left(k\right)}=\frac{Area\left(B_{t}^{\left(k\right)}\cap L_{t}\right)}{Area\left(B_{t}^{\left(k\right)}\right)},$$

where, when $R_{t}^{\left(k\right)}\geq \tau$, the target is classified as a “suspected intrusion” in the current frame. This system defaults to threshold τ=0.2 to balance detection sensitivity between minor borderline cases and substantive encroachments.

(5) Temporal Constraint: N-Frame Counting Debounce (Temporal Counter Filter)

Due to jitter and occasional false detections in single-frame detection, this paper maintains a counter $c^{\left(k\right)}$ for each target. If the t-th frame satisfies $R_{t}^{\left(k\right)}\geq \tau$, then $c^{\left(k\right)}\leftarrow c^{\left(k\right)}+1$; otherwise, $c^{\left(k\right)}\leftarrow 0$. When the counter reaches the consecutive confirmation threshold N, a final alarm is triggered:

(5) $$c^{\left(k\right)}\geq N\Rightarrow Alarm=1,$$

In our experiments, we set N=3 by default to filter out transient boundary-crossing signals caused by brief vehicle oscillations or camera jitter. If frame indices are unavailable (e.g., single-image inference), the rule degenerates to a single-frame decision N=1.

(6) Evidence Enhancement: License Plate Perspective Correction and Character Recognition (PaddleOCR)

When an alert is triggered or evidence output is required, perform perspective transformation correction on the license plate area based on the four corner points of the rotation box. This maps tilted/distorted license plates into a regular rectangular ROI. Subsequently, input the ROI into PaddleOCR (PP-OCRv4 Server + direction classification) to obtain character sequences and confidence scores:

(6) $${LP}_{t}\to {ROI}_{t}\to \left(s_{t}\;,\;p_{t}\right),$$

where $s_{t}$ represents the character recognition result, and $p_{t}$ denotes the confidence level. The system ultimately outputs “violation determination + license plate result + evidence screenshot,” forming a verifiable evidence loop.

### 3.2. Improved YOLOv5n-OBB Rotated Object Detection Model

This improved YOLOv5n-OBB model is used as the license plate detector in our cascade. The vehicle detector is implemented separately and outputs axis-aligned vehicle boxes, on which the plate detector is applied. In drone aerial images, license plates often exhibit significant rotational characteristics due to arbitrary shooting angles. Using horizontal bounding boxes (HBBs) to localize tilted license plates often introduces substantial background noise, which degrades subsequent OCR recognition. To address this, this study constructs a hybrid model optimized for Oriented Object Detection (OBB).

#### 3.2.1. “Grafting” Design of Model Architecture

Although YOLOv8 and YOLOv11 provide native OBB support, preliminary experiments in this study revealed that YOLOv8n-OBB tends to trigger early stopping on specific small-sample custom datasets, resulting in insufficient feature extraction. In contrast, the backbone networks of the YOLOv5 series demonstrated superior training stability. Therefore, we adopt a hybrid design that retains the training stability of the lightweight YOLOv5n backbone while integrating an Anchor-Free OBB head (as used in YOLOv8-style detectors).

1. Feature Extraction Backbone

The backbone of the model adopts the classic configuration from YOLOv5n, with core components including the following:

Replacement layer for the focus structure: This utilizes a 6×6 convolutional layer as the stem layer, reducing computational load while preserving the original feature information of the image.

C3 Module: This is the core component of CSPDarknet53, comprising three standard convolutional layers and multiple bottleneck structures. Unlike YOLOv8′s C2f module or YOLOv11′s C3k2 module, the C3 module features more mature inference optimization for edge devices. This model stacks 3, 6, 9, and 3 C3 modules at different feature layer depths to extract multi-scale semantic features.

SPPF (Spatial Pyramid Pooling-Fast): Positioned at the end of the backbone network, it fuses multi-receptive field features through 5×5 max pooling operations, enhancing the model’s adaptability to license plates of varying scales.

2. Rotating Detection Head (OBB Head)

We replaced the original YOLOv5 Detect head, which predicts (x,y,w,h) coordinates, with the OBB detection head from the Ultralytics framework. This head structure performs feature fusion based on PANet (path aggregation network) and adopts an Anchor-Free prediction paradigm. It directly regresses the center-point offset, scale, and rotation angle of the object, eliminating the complex anchor box clustering process inherent in traditional Anchor-Based methods.

**Figure 2:**
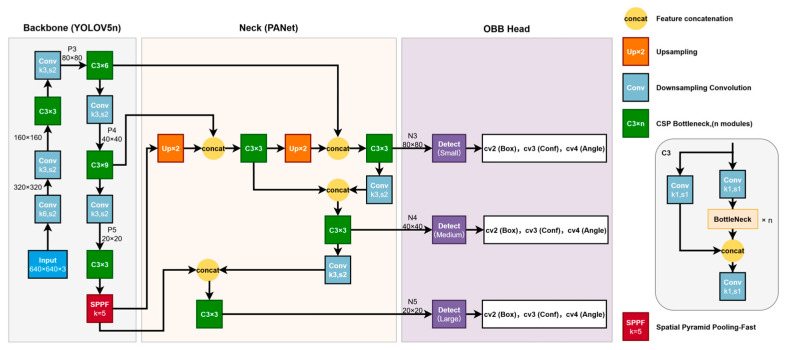
Improved YOLOv5n-OBB network topology diagram.

Figure 2 illustrates the topology of the improved YOLOv5n-OBB detection network proposed in this paper. The backbone retains YOLOv5n’s feature extraction paradigm, extracting multi-scale semantic features through stacked multi-stage C3 modules and subsampling. Multi-scale contextual fusion is achieved via SPPF at the end. The neck combines a path aggregation network (PAN) and a feature pyramid network (FPN), performing upsampling, concatenation, and re-fusion of multi-scale feature maps at different scales to enhance representation capabilities for objects of varying sizes. For the detection head, this paper introduces an Anchor-Free OBB detection head design (inspired by YOLOv8-style detection heads). It explicitly decomposes outputs at each scale into branches for bounding box regression, confidence scores, and rotation angles, enabling direct prediction of rotated bounding boxes for more precise localization of tilted license plates. By grafting the “YOLOv5n backbone + Anchor-Free OBB detector head” architecture, the model balances lightweight efficiency with robust detection capabilities for rotated objects.

#### 3.2.2. Rotation Box Definition and Mathematical Expression

To standardize rotation angle prediction, this study adopts the Long-Edge Definition method. Under this definition, the rotation bounding box is described by a quintuple:

(7) $$B=(x_{c},y_{c},w,h,\theta ),$$

Here, $\left(x_{c},y_{c}\right)$ denotes the geometric center coordinates of the bounding box. w represents the length of the rectangle’s long side, while h denotes the short side length (i.e., w≥h). θ∈[0,π) indicates the angle between the long side and the positive direction of the image coordinate system’s x-axis (in radians). This definition eliminates ambiguity in angle prediction, enabling the model to output a unique rotational pose and providing accurate geometric parameters for subsequent image correction.

#### 3.2.3. Distribution Focal Loss and Multi-Task Loss Function

To achieve high-precision localization and classification, the model’s total loss function $L_{total}$ is composed of a weighted combination of bounding box regression loss, distribution focal loss, and classification loss:

(8) $$L_{total}=\lambda_{box}L_{Rotated\_CIoU}+\lambda_{dfl}L_{DFL}+\lambda_{cls}L_{BCE},$$

1. Rotated IoU Loss ($L_{Rotated\_CIoU}$)

Traditional IoU calculations cannot handle overlapping areas of rotated rectangles. This study adopts ProbIoU Loss or Rotated CIoU Loss [47], optimized for OBBs. This loss function not only accounts for overlapping area but also introduces penalty terms for center-point distance and aspect ratio, effectively addressing gradient vanishing issues during early training stages. In this study, the weighting factor is set to $\lambda_{box}=7.5$, indicating that geometric localization accuracy dominates the training process.

2. Distribution Focal Loss ($L_{DFL}$)

To enhance localization accuracy for small targets (license plates), the model incorporates distribution focal loss (DFL) [28]. DFL transforms the continuous regression problem of bounding boxes into a discrete probability distribution prediction task. Specifically, the model discretizes the distance from the edge of the detection box to its center point into reg_max = 16 intervals. The predicted value $\hat{y}$ is computed as the expected value across each discrete interval:

(9) $$\hat{y}=\sum_{i=0}^{15}i\cdot P(y_{i}),$$

DFL optimizes the probability distribution $P(y_{i})$ to enable the network to focus on the uncertainty of bounding box locations, thereby producing robust predictions even under fuzzy boundaries. This weight is set to $\lambda_{dfl}=1.5$.

3. Classification Loss ($L_{cls}$)

Since this study involves only a single category—“license plate” (nc=1)—the classification task is relatively straightforward. Binary cross-entropy (BCE) loss is employed to optimize confidence predictions, with weights set to $\lambda_{cls}=0.5$.

#### 3.2.4. Weight Transfer and Training Strategy

Given the lack of official YOLOv5n-OBB pre-trained weights, this paper adopts a layer-wise transfer learning strategy:

1. Source Weight Selection: Load yolov5nu.pt. This is the weight file converted by Ultralytics to adapt YOLOv5n to the new codebase, with layer naming conventions compatible with v8/v11.
2. Transfer Execution: The code automatically matches and loads the weight parameters for the backbone and neck networks.
3. Cold Start and Fine-Tuning: Due to the OBB head’s structure (output channels include angle parameters) being fundamentally different from the original Detect head, these parameters are randomly initialized.
4. Training Configuration: The model is fine-tuned end to end with 100 epochs and an early-stopping patience of 10. The input resolution is set to 640×640. Data augmentation includes Mosaic (probability 1.0), HSV (Hue, Saturation, and Value) transformation, and random translation (translate = 0.1). Mosaic augmentation is disabled during the last 10 epochs to improve localization accuracy.

### 3.3. U-Net-Based Semantic Segmentation of Non-Motorized Lane

Accurate segmentation of non-motorized lane areas serves as the spatial reference for determining encroachment. Considering that lane markings in real-world scenarios may be worn, obscured by tree shade, or subject to uneven lighting, traditional threshold-based segmentation or edge detection algorithms are highly prone to failure. Therefore, this study employs the U-Net fully convolutional neural network for end-to-end pixel-level semantic segmentation.

#### 3.3.1. Network Topology: U-Net

It adopts the classic symmetric encoder–decoder architecture, whose core advantage lies in simultaneously capturing contextual information and precise localization.

**Figure 3:**
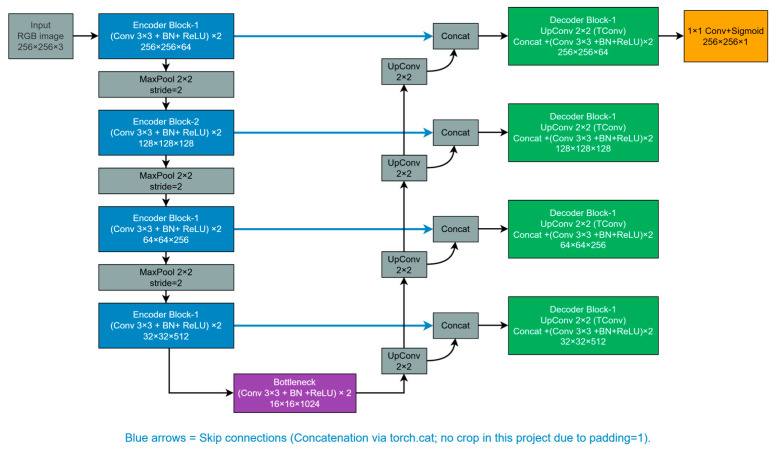
U-Net semantic segmentation network architecture diagram.

Figure 3 illustrates the U-Net semantic segmentation network architecture employed in this paper for non-motorized vehicle lane region extraction. The network adopts a classical encoder–decoder symmetric structure: the encoder comprises four downsampling stages, each containing two convolutional units, progressively reducing feature map resolution while enhancing semantic representation capabilities. The number of channels increases with depth, reaching 1024 channels at the bottleneck layer. The decoder comprises four upsampling stages. Each stage first doubles the feature map size via transposed convolution while halving the channel count, followed by skip connections (channel concatenation) with corresponding encoder layers. This compensates for spatial detail loss during downsampling and enhances boundary and contour recovery. Finally, a single-channel probability mask is output to form a binary region representing the non-motorized lane, providing pixel-level spatial constraints for encroachment detection.

1. The encoder (shrink path) normalizes the input image size to [0, 1] using ToTensor. The encoder consists of four repeated structural blocks, each containing two consecutive convolutional units. Each block contains two Convolutional (Conv)–Batch Normalization (BN)–Rectified Linear Unit (ReLU) layers, followed by a 2×2 max-pooling for downsampling. As the network deepens, the number of feature channels doubles per layer and ultimately reaches 1024 channels at the bottleneck layer. This process aims to extract high-level semantic features but sacrifices spatial resolution.
2. Decoder (Expansion Path): The decoder also consists of four structural blocks. Each block first doubles the feature map size via a 2×2 transposed convolution while halving the number of feature channels. Subsequently, a skip connection is employed to crop the feature map from the corresponding encoder layer and concatenate it with the upsampled feature map. This design effectively compensates for spatial detail information lost during downsampling, enabling the network to accurately reconstruct the edge contours of non-motorized lanes.
3. The final layer of the output layer employs a 1×1 convolution to map the feature vector into a single-channel output. This output then passes through a Sigmoid activation function, compressing the pixel values into the [0, 1] range to generate a binary mask.

#### 3.3.2. Training Optimization for Small Samples

The self-created dataset used in this study contains only approximately 800 annotated images. While the U-Net architecture itself has relatively low data requirements, to further prevent overfitting and mitigate the class imbalance issue (where non-motorized lane pixels constitute a small proportion of the entire image), this paper employs a combination of Dice Loss and BCE Loss as the loss function:

(10) $$L_{seg}=L_{BCE}+L_{Dice},$$

Dice Loss is defined as:

(11) $$L_{Dice}=1-\frac{2\sum_{i}^{N}p_{i}g_{i}+\epsilon }{\sum_{i}^{N}p_{i}^{2}+\sum_{i}^{N}g_{i}^{2}+\epsilon },$$

*p_i_* represents the predicted probability, $g_{i}$ denotes the ground truth label, and ϵ signifies the smoothing term. Dice Loss directly optimizes the overlap between the predicted region and the ground truth region, significantly enhancing segmentation completeness [48].

### 3.4. Character Recognition and Perspective Correction

Obtaining the OBB detection box for a license plate is only the first step in the pipeline. To ensure precise law enforcement, the system must clearly recognize license plate numbers. This module implements a standardized “correction–recognition” pipeline.

#### 3.4.1. Geometric Projection-Based Image Correction

Since the detection model outputs the four corner points of the license plate OBB, this system first sequentially normalizes the corner points (top left (TL), top right (TR), bottom right (BR), and bottom left (BL)). It then estimates the target width and height (W,H) based on edge lengths, constructs a homography matrix H=getPerspectiveTransform(src,dst), and uses warpPerspective to remap the tilted license plate region into a horizontal rectangular ROI. This matrix is used to perform perspective correction on the original license plate region, remapping the skewed non-rectangular area into a horizontal rectangular image. This step eliminates geometric distortion interference with the OCR model.

**Figure 4:**
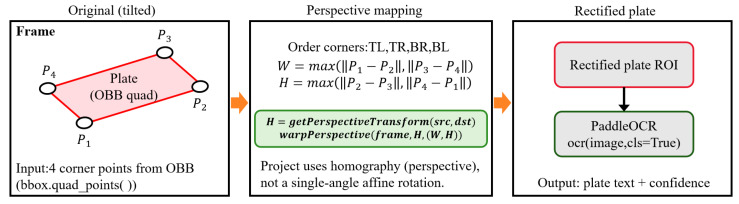
Schematic diagram of license plate perspective transformation correction.

Figure 4 illustrates the processing flow of this system after obtaining license plate detection results, performing perspective correction on the license plate region, and completing character recognition. The left side shows a schematic of the original video frame, where license plates typically exhibit tilt and perspective distortion. The detection module outputs the OBB corner points (P1–P4) of the license plate, used to precisely describe the rotation and deformation boundaries of the license plate. The central section details the core steps of geometric correction: First, the corner points undergo sequential normalization (TL, TR, BR, and BL), and the width and height $\left(W,H\right)$ of the target rectangle are estimated based on the four side lengths. Subsequently, the homography matrix H is constructed (getPerspectiveTransform(src,dst)), and warpPerspective maps the tilted license plate region onto a horizontal rectangular region of interest (ROI). The right panel displays the corrected license plate image and OCR processing: the rectified ROI is fed into PaddleOCR (with orientation classification enabled: cls = True), outputting recognized characters and their confidence scores. This workflow leverages OBB quadrilateral information to eliminate perspective distortion, thereby enhancing OCR recognition stability and accuracy in complex scenarios such as tilted shots and varying viewing angles.

#### 3.4.2. PP-OCRv4 Server Edition Recognition Model

Considering that drone images may suffer from motion blur or insufficient resolution, the lightweight mobile model (Mobile) may fail to meet recognition accuracy requirements. Therefore, this system integrates the PP-OCRv4 Server Edition model from the PaddleOCR framework. This model comprises three cascaded sub-networks:

1. Text Detection (Detection): This utilizes the ch_PP-OCRv4_det_infer model, employing the DBNet (Differentiable Binarization) algorithm to precisely locate and correct character regions within calibrated images.
2. Orientation Classification (Classification): This uses the ch_ppocr_mobile_v2.0_cls_infer model to determine whether license plates are rotated 180° and automatically corrects them.
3. Text Recognition: This utilizes the ch_PP-OCRv4_rec_infer model. Based on the SVTR (Scene Text Recognition with a Single Visual Model) architecture, this model integrates CNN’s local feature extraction capabilities with Transformer’s global sequence modeling capabilities, enabling effective recognition of mixed sequences of Chinese characters, letters, and numbers [49].

### 3.5. Logic for Determining Encroachment and Spatial–Temporal Constraints

To transform visual perception results into legally valid evidence of violations, this study designed a rigorous logical determination algorithm. This algorithm abandons simple “line-crossing detection” in favor of a more robust “area overlap analysis.”

#### 3.5.1. Area Ratio Criterion

We first define the axis-aligned vehicle bounding box in frame t as $V_{t}$ and define the lane region polygon obtained from U-Net as $L_{t}$. The degree of vehicle intrusion into the non-motorized vehicle lane is quantified by the intrusion ratio $R_{t}$:

(12) $$R_{t}=\frac{Area(B_{t}\cap L_{t})}{Area(B_{t})},$$

This formula calculates the ratio of the intersection area between the vehicle bounding box and the non-motorized lane area to the total vehicle area. Threshold setting: Extensive experimental verification has established an encroachment threshold of τ=0.2. That is, when more than 20% of a vehicle’s area enters the non-motorized lane, the system flags the frame as a potential violation. This threshold effectively filters out minor edge occlusions caused by perspective projection while ensuring recall rates for substantive encroachment incidents.

#### 3.5.2. Temporal Counter Filter

Single-frame detection often suffers from uncertainties (e.g., detection box jitter and mask edge flickering). To avoid false positives, the system introduces spatio-temporal consistency constraints.

Target tracking: The system prioritizes using track_id (integrated with the ByteTrack tracker) to lock onto the same vehicle.

State machine logic: One must maintain a counter *C* for each target. If the current frame R>τ, then C←C+1. If the current frame R≤τ, then C is reset to 0.

Alarm triggering: The system triggers a final alarm only when $C\geq N_{thresh}$ (where $N_{thresh}=3$ in this system), meaning the target is determined to be in violation for three consecutive frames.

**Figure 5:**
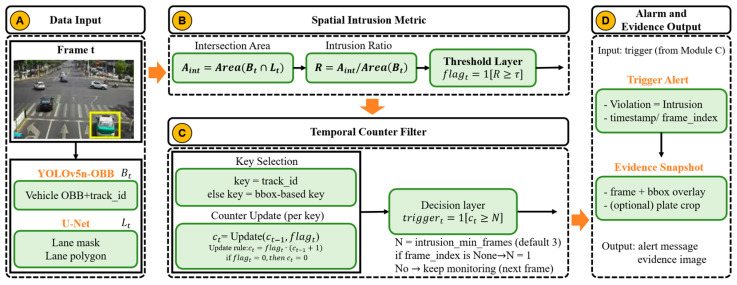
Violation determination logic flowchart. In panel A, the yellow box marks the target vehicle instance whose spatial intrusion ratio is subsequently evaluated.

As shown in Figure 5, to suppress jitter and occasional false positives caused by single-frame detection, the system introduces a counter debouncing mechanism based on temporal consistency. For each input frame, the system first acquires the vehicle detection box and the non-vehicle lane area (extracted from segmentation results as lane polygons) and then calculates the overlap ratio R between the vehicle box and the lane area. When R≥τ (where threshold τ is defined by the system configuration intrusion_iou_threshold, default 0.2), the target is classified as a “suspected intrusion,” incrementing the corresponding counter by 1; otherwise, the counter is reset. Counters are maintained using target keys, with the system prioritizing track_id output from detectors to associate vehicles. When track_id is unavailable, the system falls back to a temporary key constructed from vehicle bounding box coordinates to ensure process viability. Only when the counter reaches the consecutive confirmation threshold *N* (defined by the intrusion_min_frames configuration, default 3) does the system trigger a final alert and save screenshot evidence; if the threshold is not met, it continues waiting for subsequent frame updates to increment the counter. This mechanism filters out short-term jitter and boundary flickering through “consecutive frame confirmation,” enhancing the stability and reliability of alarm outputs. (In scenarios where frame_index is not provided, the system treats the consecutive confirmation threshold as *N* = 1, meaning multi-frame debouncing constraints are disabled.)

This mechanism ensures the stability of the alarm system, effectively reducing transient false alarms caused by momentary attitude adjustments of the drone and thereby significantly enhancing the system’s practicality.

## 4. Experimental Results and Analysis

### 4.1. Experimental Setup

This section systematically evaluates each module of the proposed visual warning system for motor vehicles encroaching on non-motorized lanes through a series of comparative and ablation experiments. All experiments were conducted under identical hardware and software conditions to ensure fairness and reproducibility of results.

This section’s experiments primarily utilize three datasets:

1. The self-built Drone dataset, containing multiple top-down and oblique perspectives;
2. The publicly available Chinese City Parking Dataset (CCPD) of license plate data for standard street scene comparisons;
3. The BDD100K dataset for non-motorized lane semantic segmentation experiments.

Implementation details: Experiments were conducted on a workstation equipped with an NVIDIA GeForce RTX 4090 graphics processing unit (GPU). The software environment comprised Python 3.10, PyTorch 2.1.0, CUDA 12.2, Ultralytics 8.0.196, and PaddleOCR 2.7.0.3. Detection was implemented using PyTorch/Ultralytics, and OCR was implemented using PaddleOCR. Key training hyperparameters and system decision parameters (e.g., τ and *N*) are summarized in Table 1.

BDD100K dataset: BDD100K is used for pre-training the segmentation backbone with road/sidewalk labels. We follow the official BDD100K train/validation split and fine-tune the model on the drone lane mask annotations for the final lane region segmentation used in our system.

CCPD dataset: CCPD is used as auxiliary data for license plate appearance diversity. We follow the official split/protocol of CCPD and report results on our Drone test set when evaluating the end-to-end enforcement pipeline.

Drone dataset: The self-collected Drone dataset contains approximately 800 annotated images/frames (about 500 from UAV perspectives and the rest from high-mounted cameras) covering multiple intersections, different flight heights, tilt angles, lighting conditions, partial occlusions, and borderline encroachment cases. Each frame is annotated with (i) vehicle horizontal bounding boxes for occupancy computation, (ii) license plate oriented bounding boxes (four-point quadrilaterals) for evidence extraction, and (iii) a binary mask for the enforceable non-motorized lane region. The samples were manually annotated by Tian He and Weishen Wu and then verified in a second-pass quality-control review. Ambiguous cases—especially lane boundaries near curbs, partially occluded vehicles, and rotated plate corners—were resolved by consensus with reference to the original full-resolution frames. To reduce temporal leakage, we adopt a scene-wise split by video sequence and intersection: frames from the same video clip are kept in the same split. Unless otherwise stated, we use a 70/15/15 split for train/validation/test. Representative examples from each split were re-checked before training to verify label consistency and to ensure that no video clip was shared across train, validation, and test partitions.

### 4.2. Recognition Performance Comparison of Different OCR Methods

License plate character recognition is the core step in this system’s violation evidence collection process. To balance recognition accuracy and real-time performance in edge computing environments, this experiment aims to compare the performance of different OCR engines in complex surveillance scenarios, thereby selecting the most suitable text recognition solution for this system.

Based on unified license plate detection results (YOLOv5n-OBB), we compare three OCR methods: LPRNet, EasyOCR, and PaddleOCR. All OCR models are evaluated on cropped and perspective-rectified license plate ROIs rather than full video frames. In the deployed system, OCR is invoked only when a candidate violation is detected (evidence-trigger processing), so the end-to-end system throughput is dominated by detection and segmentation during normal (non-alarm) frames.

Character Accuracy (CA): This measures the proportion of individual characters correctly recognized in the output.

Plate Accuracy (PA): This only counts as correct when all characters within the license plate are recognized accurately, serving as a core metric for system reliability.

Inference Speed (FPS): This is the number of image frames processed per second, used to evaluate the algorithm’s real-time response capability at the edge to assess its suitability for license plate recognition tasks from a drone perspective.

Average Inference Time: This metric is inter-related and complementary to inference speed (FPS), quantifying the processing time required for a single license plate image. It serves as a direct parameter for evaluating the algorithm’s real-time response capability at the edge.

The recognition performance comparison of different OCR methods is shown in Table 2.

Analysis of the experimental data (as shown in Table 2) reveals that PaddleOCR significantly outperforms the other two approaches in both character-level accuracy and overall license plate recognition rate. It demonstrates particularly stable performance in recognizing Chinese provincial characters and handling tilted license plates. Although LPRNet achieved an FPS of 117.96—5.3 times that of PaddleOCR—its low recognition rate for Chinese provincial characters (only 78%) and tendency to confuse similar characters (e.g., 0/O, 1/I) resulted in a total plate recognition rate (PA) of merely 40.00%. This performance falls short of meeting stringent traffic enforcement requirements. As a general-purpose OCR engine, EasyOCR ranks last in both CA and PA metrics for license plate recognition, with an inference speed of only 8.44 FPS, failing to meet the real-time requirements for intersection monitoring.

In summary, while LPRNet achieves higher raw OCR throughput, the YOLOv5n-OBB + PaddleOCR solution provides the highest recognition accuracy among the compared OCR methods and improved robustness to plate tilt and complex backgrounds, which is critical for generating reliable enforcement evidence. Note that the FPS reported in Table 2 is measured on cropped license plate ROIs for the OCR module, whereas the end-to-end system FPS (with detection, segmentation, and recognition) is reported later together with the full system ablation results.. Therefore, subsequent experiments uniformly adopt PaddleOCR as the license plate character recognition module.

### 4.3. Performance Comparison of OBB Detection Models

For license plates—rigid objects with fixed geometric shapes and strong edge features—this study compares the adaptability of mainstream single-stage detectors against the proposed YOLOv5n-OBB hybrid model. While the proposed license plate detector is trained for the license plate class, this subsection focuses on the plate class because it is the more challenging small object and directly affects OCR evidence quality.

The experiment utilized a custom-built private license plate OBB dataset. Training was conducted uniformly at an input resolution of 640×640, with a batch size of 32 and a total of 100 training epochs. An early stopping mechanism with a tolerance of 10 epochs was implemented to prevent overfitting.

The core evaluation metrics for the experiment include mean average precision (mAP50 and mAP50-95), precision, recall, validation box loss, and state. The detection performance comparison across different models is shown in Table 3.

Table 3 shows that YOLOv5n-OBB achieves 86.6% on the stringent mAP50-95 metric, representing a 5.8 percentage point improvement over the official baseline YOLOv8n-OBB. This indicates that the rotation-predicted boxes generated by this hybrid architecture exhibit exceptional geometric alignment with actual license plates. YOLOv8n-OBB triggered early stopping at the 54th epoch, and its validation set BoxLoss (0.591) was significantly higher than that of YOLOv5n-OBB (0.482). This suggests that the complex C2f module may introduce redundant information when processing relatively feature-poor license plate data, causing the model to get stuck in local optima. In contrast, the training process of YOLOv5n-OBB demonstrated greater robustness. Furthermore, while maintaining high accuracy, the model developed in this study achieved a recall rate of 91.1%, effectively reducing the false negative rate in complex environments (e.g., large-angle tilts and blurred edges).

**Figure 6:**
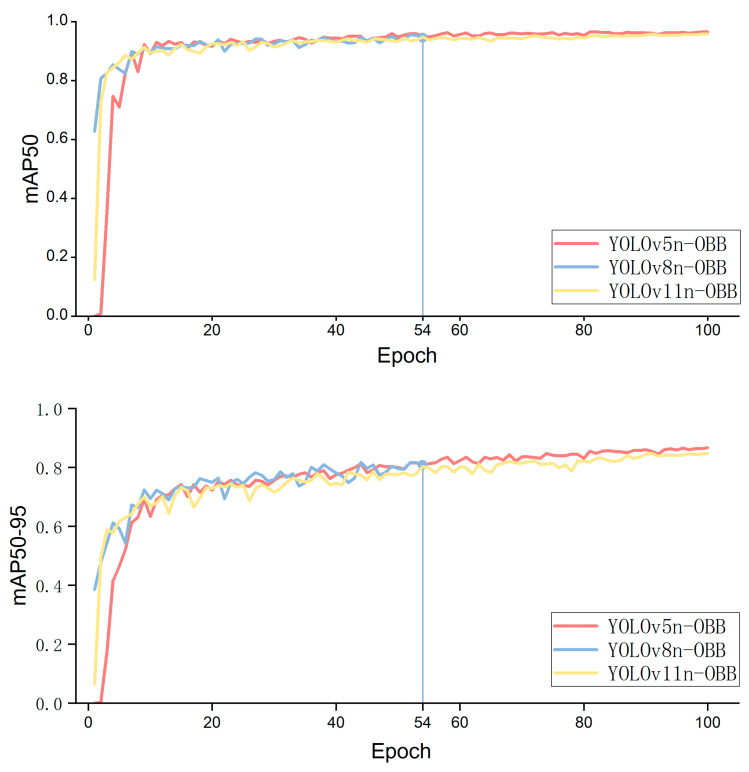
Comparison of mAP curves during training.

Analysis of Table 3 and Figure 6 indicates that YOLOv5n-OBB provides a favorable balance between overall accuracy and recall. Although YOLOv8n-OBB achieves slightly higher precision metrics in certain aspects, it exhibits premature stopping during training, resulting in its overall performance failing to reach its full potential.

### 4.4. Cross-View Generalization Evaluation

In real-world traffic monitoring scenarios, the installation height, downward tilt angle, and lateral angle of cameras are often random, requiring detection models to possess strong viewpoint generalization capabilities. This experiment aims to investigate the contribution of different data sources to the model’s learning of rotation-invariant features by comparing the effects of training with the standard street-view dataset (CCPD) and the multi-angle drone-view dataset (Drone). Additionally, cross-domain cross-validation is employed to analyze the model’s environmental adaptability.

All experiments were conducted based on the YOLOv5n-OBB architecture, trained for 50 epochs on a single GPU. To evaluate the model’s generalization capability across different perspective data distributions, four sets of comparative studies were designed, encompassing both intra-domain self-testing and cross-domain cross-validation modes. The cross-validation experimental design is shown in Table 4.

**Figure 7:**
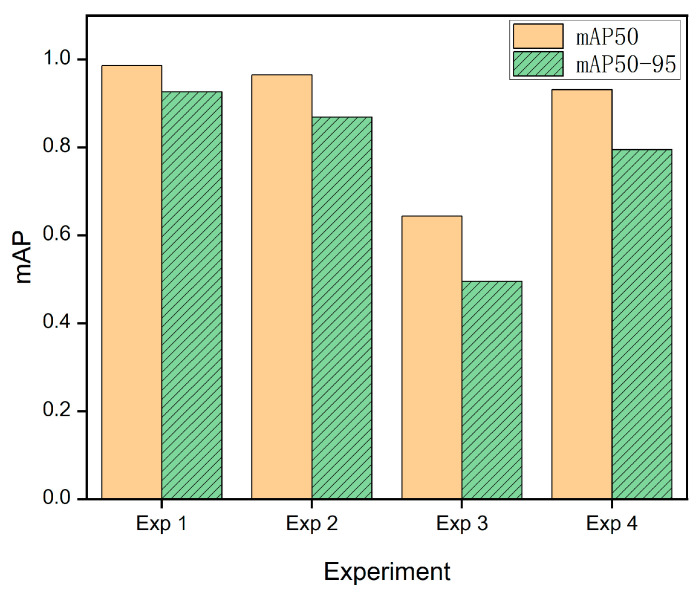
Bar chart comparing mAP across four experimental groups.

By analyzing Table 5 and Figure 7, in Exp1, the CCPD dataset features a single perspective and highly standardized license plate characteristics, enabling the model to achieve high in-domain performance (mAP50-95 > 0.92). In Exp2, despite the Drone dataset containing numerous complex perspectives such as top-down and oblique views, the model maintained an mAP50 of 0.965, demonstrating the model’s robustness to complex rotated objects. However, when models trained solely on standard street scenes encountered drone data with large angles, mAP plummeted to 0.644. This indicates that the training set, predominantly composed of “level or slightly downward” samples, trapped the model in a local optimum for perspective, preventing it from learning truly rotation-invariant features. When confronted with extreme geometric distortions, the model exhibited frequent false negatives and severe bounding box jitter. Exp4 demonstrates impressive generalization performance (mAP50 = 0.931). This is mainly because the Drone dataset covers a broader range of viewpoints, rotations, and background variations than CCPD, thereby exposing the model to richer geometric transformations and clutter. After training under this more challenging data distribution, the model handles simple street-view data with ease.

In summary, models trained solely on street-view data exhibit significant performance degradation under drone perspectives, indicating inadequate modeling capabilities for large-angle rotations and top-down features. Conversely, models trained on drone data maintain high accuracy in complex perspectives while still performing stably on street-view data, indicating better cross-view generalization than the street-view-only training setting.

Implications for the end-to-end enforcement task: The above cross-perspective results indicate that training with only street-view data (CCPD) is insufficient for UAV or high-mounted views, which directly affects license plate localization and thus the quality of OCR evidence. In the proposed end-to-end system, we therefore prioritize multi-view training (Drone + CCPD) and adopt OBB detection to explicitly model large rotations. This improves the reliability of the plate evidence branch under cross-view conditions, while the deployed encroachment decision remains HBB based, as described in Section 3.1.2.

### 4.5. Performance Evaluation of Lane Region Segmentation

In traffic violation detection systems, accurately delineating non-motorized vehicle lane boundaries serves as the physical reference for determining encroachment. This study employs a U-Net semantic segmentation model that is pre-trained on the large-scale BDD100K driving dataset and then fine-tuned for the deployed lane region task on the Drone dataset. This dataset encompasses diverse complex scenarios including urban and suburban environments, featuring exceptionally high environmental diversity.

In the deployed system, the enforceable non-motorized lane region is defined by our manually annotated binary masks on the Drone dataset; the road/sidewalk labels from BDD100K are used only for pre-training to learn generic boundary features.

This experiment evaluates the U-Net segmentation module used to extract the enforceable non-motorized lane region. To improve generalization under small-sample UAV annotations, we adopt a two-stage training strategy: (i) pre-training on the public BDD100K semantic segmentation labels (road/sidewalk) to learn generic drivable-boundary features and (ii) fine-tuning on our self-collected Drone dataset where the non-motorized lane region is manually annotated as a binary mask. For qualitative visualization, we report representative examples that contain the sidewalk label to enable a meaningful comparison between ground truth and predictions.

In the pre-training stage, the network is trained with two categories (“road” and “sidewalk”) from BDD100K. In the fine-tuning and evaluation stage (used by the proposed system), the network outputs a single-channel binary mask indicating the non-motorized lane region. The mask is post-processed by connected-component filtering and contour extraction to obtain a polygonal lane region for subsequent area intersection computation.

The experiment was tested on 100 validation samples, with the model’s total parameters amounting to approximately 31.04 million. Specific performance data are presented in Table 6.

As shown in Table 6, the segmentation module achieves a recall of 72.35%, which helps avoid missed alarms caused by under-segmentation of the lane region. The moderate precision (46.13%) and IoU (39.63%) mainly stem from boundary ambiguity in aerial views (shadows, worn lane markings, and weak contrast near curbs). Importantly, the downstream decision does not rely on per-pixel perfection: the encroachment coefficient is computed from polygon-level intersection between the vehicle bounding box (axis-aligned) and the lane region polygon, so small boundary deviations typically cause only minor changes in the overlap ratio. Moreover, the threshold sensitivity analysis in Section 4.9 shows that the final violation decision remains stable across a practical range of thresholds.

**Figure 8:**
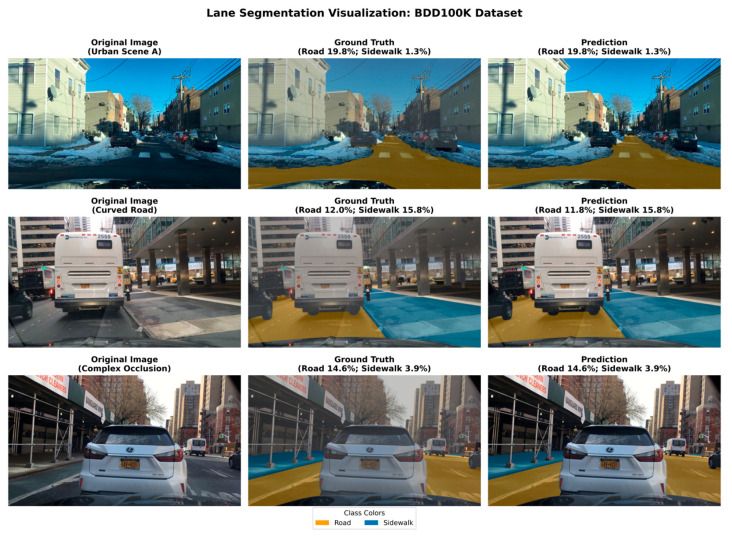
Lane region segmentation visualization on BDD100K. Each row shows the original image (**left**), ground truth mask (**middle**), and prediction mask (**right**). Road regions are highlighted in yellow and sidewalk regions in blue. The percentage of pixels assigned to each class (Road x%; Sidewalk y%) is reported in the titles for both ground truth and predictions to facilitate a quantitative visual comparison.

As shown in Figure 8, the U-Net model produces visually consistent lane region masks across diverse BDD100K scenes. To avoid ambiguity caused by scenes that contain negligible sidewalk regions, we present three representative samples that include the sidewalk label. The overlays indicate that the model captures the overall extent of both road and sidewalk regions, and the reported pixel percentages for Road (x%) and Sidewalk (y%) show that the predicted class proportions closely follow the ground truth in these examples. On urban straight roads, the predicted boundaries are coherent and preserve the main road structure. For curved or perspective-distorted scenes, the model remains spatially adaptable and maintains reasonable separation between road and sidewalk. In complex cases with partial occlusions by large vehicles, the predicted masks may exhibit slightly blurred boundaries, yet they still recover the dominant drivable region and preserve the dominant region structure sufficiently for qualitative boundary assessment during pre-training analysis.

In summary, although segmentation accuracy in extremely complex scenarios still has room for improvement, the current U-Net model provides sufficient spatial support for the downstream encroachment decision under UAV viewpoints.

### 4.6. Controlled Benchmark Evaluation of Encroachment Decision Methods

To further isolate the geometric contribution of the decision representation and temporal filtering, we next present a controlled benchmark that is intentionally simplified relative to the final deployed pipeline. This subsection is a controlled analysis of HBB-vs.-OBB occupancy estimation and is not identical to the final deployed G4 pipeline. It is intended to illustrate the geometric effect of box representation and temporal filtering in challenging encroachment scenarios. The experiment focuses on comparing the performance differences between traditional and improved approaches when handling critical scenarios such as vehicle tilting and lane-crossing violations.

The experiment set up two groups for comparison. The first group is the baseline, which uses YOLOv5n (HBBs, horizontal bounding boxes) for detection, combined with a vehicle center-point falling-into-region method for classification, without any temporal processing logic. The second group is a controlled OBB-reference scheme for geometric comparison. It uses an area ratio method (with a threshold of 20%) for classification and incorporates a three-frame sequential filtering logic. The test set comprises 100 representative samples covering straight-line encroachment, diagonal cutting in, critical lane-crossing, normal driving, and brief boundary interaction. We treat this benchmark as a representative pilot-style evaluation set rather than a definitive large-scale deployment benchmark. To mitigate the limited sample size, we additionally report Wilson 95% confidence intervals, scenario-wise breakdowns, and a complementary temporal stability evaluation on 20 video clips (200 frames). Table 7 presents the scenario-wise breakdown on the same 100-sample evaluation set.

Table 7 additionally reports the positive and negative sample counts for each subset to clarify the class distribution behind the scenario-wise results. These subsets are intended to illustrate representative scene conditions rather than to form class-balanced partitions.

The performance comparison of intrusion behavior recognition through automated testing of 100 samples is shown in Table 8.

Experimental results show that the controlled OBB-reference scheme outperforms the baseline method in terms of accuracy, recall, and F1-score. On the 100-sample test set (66 encroachment cases and 34 normal cases), the controlled OBB-reference scheme achieves 92.0% accuracy (Wilson 95% confidence interval (CI): 85.0–95.9%), compared with 67.0% (95% CI: 57.3–75.4%) for the baseline. The false positive rate is reduced from 32.35% (95% CI: 19.1–49.2%) to 5.88% (95% CI: 1.6–19.1%). The improvement in recall (+24.24%) compensates for the traditional center-point method’s tendency to miss violations when vehicles drive along lane boundaries, and the lower FPR provides more stable alarms in oblique-entry and borderline scenarios. To provide a more complete evaluation under class imbalance, we additionally report balanced accuracy and MCC in Table 8. Both metrics consistently support the superiority of the controlled OBB-reference scheme: balanced accuracy improves from 67.16% to 92.51%, and MCC increases from 32.68% to 82.98%.

**Figure 9:**
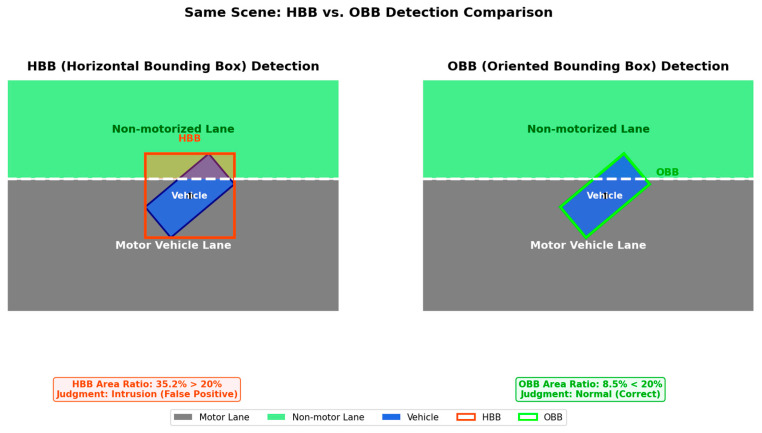
Schematic diagram comparing HBB and OBB detection in the same scene.

To more clearly illustrate the geometric effect of HBB-versus-OBB occupancy estimation in oblique driving scenarios, Figure 9 compares two box representations in the same scene:

As shown in Figure 9, HBBs may include redundant background in diagonal-driving cases, which can inflate the overlap ratio near the lane boundary. The OBB example is shown only as a geometric reference; the deployed online system still uses HBB-based occupancy estimation.

**Figure 10:**
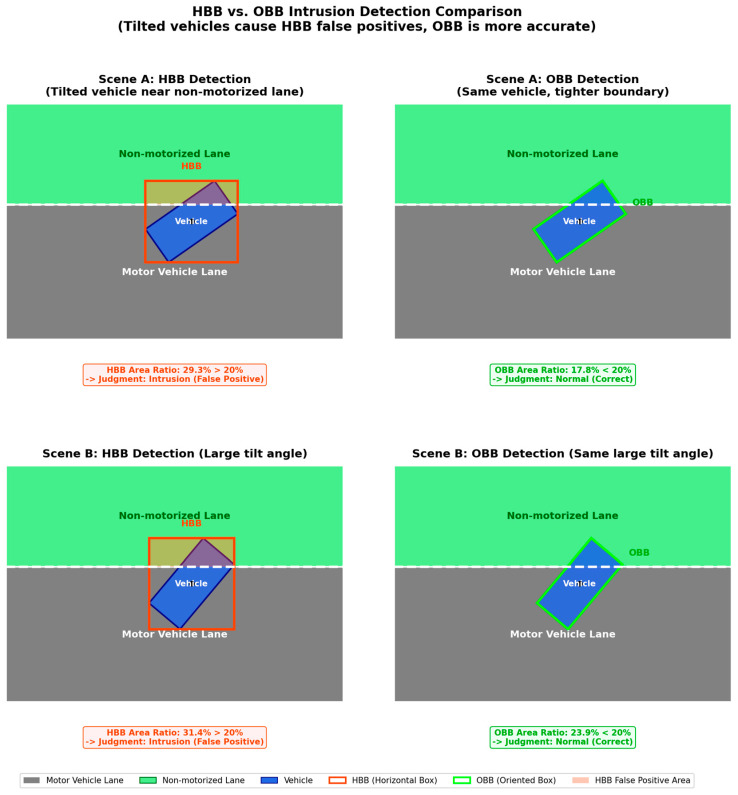
Comparison of determinations at different inclination angles (Scene A: 35°; Scene B: 50°).

Further scene generalization tests, as shown in Figure 10, demonstrate detection performance for vehicles tilted at large angles of 35° and 50°:

Figure 10 further illustrates that as vehicle tilt increases, HBB-based overlap estimation becomes more sensitive to redundant background coverage. This effect motivates the controlled OBB reference used here for geometric interpretation.

In critical lane-crossing scenarios, the area ratio method allows for defining violation severity by setting precise percentage thresholds (set to 20% in this system), offering greater flexibility than single-center-point detection. For transient lane-crossing events, implementing a three-frame temporal filter enables the system to eliminate false signals caused by evasive maneuvers or brief vehicle oscillations. This temporal filtering is the key technical mechanism driving the false alarm rate reduction from 32.35% to 5.88%.

In summary, the controlled comparison shows that orientation-aligned geometry helps explain difficult oblique-entry cases, while the deployed system addresses practical stability through the HBB-based decision pipeline evaluated in the following subsections.

### 4.7. System Component Ablation Experiment

To quantitatively analyze the contributions of rotation detection (OBB), semantic segmentation (U-Net), and advanced OCR (PaddleOCR) to the system’s overall recognition accuracy and real-time performance, we decomposed the core components into four experimental groups: G1 serves as the baseline (vehicle HBB detector + fixed ROI mask + LPRNet), G2 adds rotation detection (vehicle HBB detector + plate OBB detector (for evidence crops) + fixed ROI mask + LPRNet) to G1, G3 replaces G2′s lane segmentation with U-Net segmentation (vehicle HBB detector + plate OBB detector + U-Net lane segmentation + LPRNet), and G4 represents the final project version, substituting G3′s character recognition with PaddleOCR (vehicle HBB detector + plate OBB detector + U-Net lane segmentation + PaddleOCR).

Performance comparisons under different system configurations are shown in Table 9.

**Figure 11:**
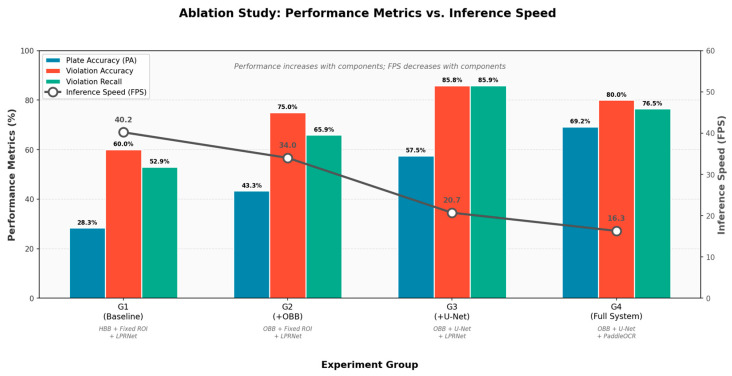
Comparison of ablation experiment performance and FPS.

Table 9 shows that OBB rotation detection and U-Net dynamic segmentation make the largest contributions to system performance improvement, while PaddleOCR primarily improves the reliability of evidence for final license plate recognition. Although the complete system experiences a slight decrease in inference speed, it remains usable for near-real-time alerting on the target hardware. Figure 11 visually illustrates the upward trend of performance metrics with component stacking and the corresponding increase in computational overhead through a dual-axis graph. After introducing OBB (G1→G2), violation detection accuracy improved from 60.0% to 75.0%, while the license plate recognition rate increased from 28.3% to 43.3%. This demonstrates that rotating bounding boxes enable more precise localization and reduce background interference in oblique views. After introducing U-Net (G2→G3), violation detection accuracy further increased to 85.83%, while recall improved from 65.88% to 85.88%. This indicates that dynamic lane segmentation adapts to complex intersection geometries and reduces misclassifications caused by inaccurate lane localization. Switching to PaddleOCR (G3→G4) achieved the highest plate recognition accuracy (PA) of 69.17%. G4 is optimized for evidential reliability rather than pure violation classification score. In this configuration, OCR and evidence output are triggered only for candidate violations, and low-quality plate crops are filtered before evidence generation. This slightly lowers violation classification metrics relative to G3 but yields substantially more reliable plate evidence for enforcement-oriented use. Although the increased model complexity reduces FPS from 20.7 to 16.3, the system remains usable for near-real-time alerting on the target hardware. In addition, for the complete deployed system (G4), the measured end-to-end latency is p50 = 61.44 ms and p95 = 75.32 ms on the target hardware, indicating stable near-real-time performance.

### 4.8. Temporal Stability Evaluation of the Deployed HBB-Based Decision Pipeline

Beyond the component-wise ablation above, we further evaluate whether the temporal counter improves decision stability under the deployed HBB-based encroachment logic. In this experiment, 20 video sequences (200 frames in total) containing actual encroachment and borderline driving behaviors were used to compare Single-frame HBB decisions with Temporal HBB (*N* = 3).

Two control groups were established:

1. Single-frame HBB: using axis-aligned vehicle boxes with a single-frame decision as the baseline;
2. Temporal HBB (N=3): applying the same N-frame temporal counter on top of the Single-frame HBB decisions to isolate the effect of temporal filtering, where a violation must be detected in three consecutive frames to trigger an alert.

**Figure 12:**
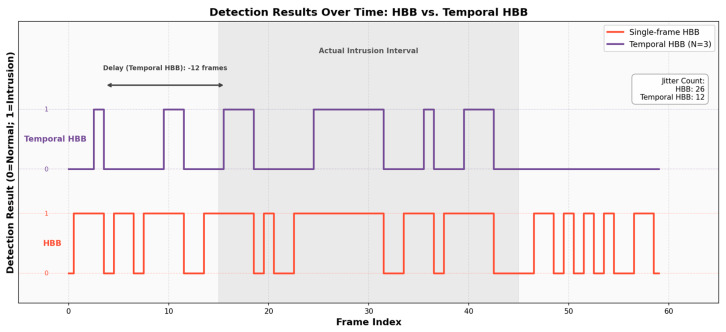
Temporal decision sequences for Single-frame HBB and Temporal HBB (*N* = 3) on a representative clip (0: normal; 1: intrusion). The shaded region indicates the actual intrusion interval.

Figure 12 shows the per-frame decision sequence (0: normal; 1: encroachment) for the baseline Single-frame HBB and the Temporal HBB (*N* = 3) strategy on a representative video clip. The Single-frame HBB baseline exhibits frequent decision oscillations near lane boundaries due to camera motion and detection noise, which can easily cross the encroachment threshold intermittently. In contrast, the Temporal HBB (*N* = 3) strategy effectively suppresses transient spikes by requiring consecutive confirmations, yielding a decision sequence that better aligns with the visually continuous intrusion interval, at the cost of a small response delay.

The experiment quantitatively analyzed the false positive rate (FPR), stability index, and response latency, with the results shown in Table 10.

These results support the use of temporal debouncing as a practical stability mechanism in the deployed HBB-based pipeline.

### 4.9. Threshold Sensitivity Analysis

In the violation detection logic, the threshold τ of the encroachment coefficient ($R_{intrusion}$) serves as the core parameter that balances detection sensitivity and false alarms. To determine a practical default, we conducted a threshold sensitivity experiment on a 200-sample set, sweeping τ in the range [0.10, 0.40] with a step size of 0.05.

**Figure 13:**
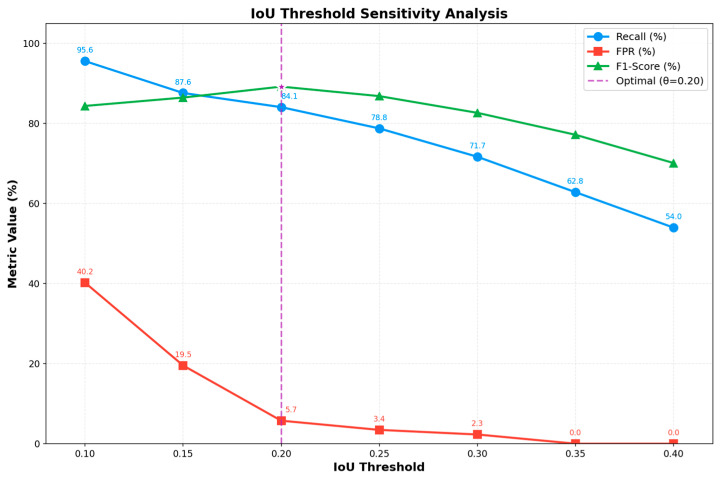
Encroachment threshold sensitivity curve.The purple dashed line and the highlighted marker indicate the optimal IoU threshold, selected as the value that maximizes the F1-score (*θ* = 0.20 in this experiment).

Threshold effects on performance metrics: The experiment meticulously documented core metrics such as recall, false positive rate (FPR), and F1-score under various threshold settings. As shown in Figure 13, each metric exhibits a pronounced trend in response to threshold variations.

As the threshold increases, recall exhibits a monotonically decreasing trend, while the false positive rate (FPR) also decreases accordingly. This indicates that higher thresholds require vehicles to occupy a larger area within the non-motorized lane. While this effectively eliminates false alarms caused by perspective projection errors, it also leads to missed detections of minor violations. The F1-score, as the harmonic mean of precision and recall, serves as a key metric for evaluating the system’s overall performance. Experimental results show that the F1-score peaks at 89.20% when the threshold is set to 0.20. At this threshold, the system maintains a high recall rate of 84.07% while suppressing the false positive rate to a low level of 5.75%, achieving an optimal balance in detection performance.

Configuration strategies for different scenarios: Based on experimental findings, this system proposes three configuration recommendations tailored to different industrial application requirements:

High Recall Priority Configuration (Threshold = 0.15): This is suitable for evidence archiving scenarios requiring maximized violation detection rates, capturing the vast majority of encroachment incidents despite potentially incurring approximately 19% manual review burden.

Standard Balanced Configuration (Threshold = 0.20): This is the default setting for this system, designed to maximize F1-score. It is suitable for the vast majority of urban intersection monitoring tasks.

Low False Positive Priority Configuration (Threshold = 0.35): This is suitable for real-time automated alert systems. At this threshold, FPR drops to 0%, ensuring every generated alert represents a substantive, severe violation and minimizing disruption to management personnel.

## 5. Conclusions and Outlook

This paper addresses the challenge of monitoring motor vehicles encroaching on non-motorized lanes at urban intersections by designing and implementing an intelligent surveillance system integrating rotating object detection and semantic segmentation. By incorporating the YOLOv5n-OBB architecture and U-Net dynamic segmentation model, the system effectively mitigates projection distortion issues associated with traditional horizontal detection boxes under oblique viewing angles. Utilizing the “area ratio method” combined with an “N-frame temporal filtering” mechanism, the false positive rate (FPR) is significantly reduced to 5.88%. Experiments demonstrate that this solution enhances detection accuracy while ensuring automated and precise license plate evidence extraction, providing a reliable technical approach for smart urban traffic governance.

Building upon this foundation, the proposed system demonstrates practical engineering utility for UAV-assisted traffic monitoring by leveraging the high-altitude flexibility of unmanned aerial vehicles (UAVs). On one hand, it overcomes the blind spot limitations of traditional fixed surveillance, transforming enforcement from inefficient “manual violation detection” to efficient “automated alerts-on-site resolution.” Through precise visual evidence collection and dynamic deterrence, it effectively curbs driver complacency and reduces enforcement disputes. On the other hand, the spatio-temporal violation data accumulated during system operation can be used to precisely identify traffic black spots. This provides scientific support for municipal departments to evaluate the effectiveness of road barriers and optimize non-motorized lane design, thereby supporting a transition from case-based enforcement toward more refined traffic governance.

However, several limitations remain. First, segmentation and OCR performance may degrade under heavy rain, dense fog, low illumination, or severe occlusion. Second, the current end-to-end validation is still limited in scale, with the main encroachment benchmark containing 100 labeled samples and the temporal stability analysis covering 20 video clips (200 frames). Although these evaluations are complemented by confidence intervals and scenario-wise breakdowns, a larger multi-intersection, multi-time-of-day benchmark is still needed to further quantify generalization. Third, because the system integrates detection, segmentation, and OCR, deployment on ultra-low-power edge devices will require further optimization of energy efficiency and latency.

Future research can be pursued in several directions: first, enhancing the system’s perception capabilities in adverse weather conditions by incorporating image enhancement algorithms or multimodal data (e.g., infrared imaging); second, exploring lightweighting techniques based on knowledge distillation or model pruning to adapt to computationally constrained embedded surveillance front ends; and finally, expanding single-point monitoring to multi-camera collaborative detection, which could leverage the spatio-temporal correlation of vehicle trajectories for more complex violation traceability analysis.

In summary, the proposed system improves spatial decision robustness and temporal stability under UAV viewpoints while providing readable plate evidence for enforcement-oriented traffic monitoring. These results demonstrate the practical feasibility of the proposed perception-to-decision pipeline for multi-view traffic governance applications.

## Figures and Tables

**Table 1:** Key hyperparameters and system parameters.

Module	Setting/Notes
OBB detection (YOLOv5n-OBB)	Input 640×640; batch size 32; 100 epochs; early stopping patience 10 (Section 4.3)
Lane segmentation (U-Net)	≈31.04 M parameters; pre-trained on BDD100K (road/sidewalk) and fine-tuned on drone lane mask annotations (Section 4.5)
OCR (PaddleOCR)	PP-OCRv4; perspective-rectified plate ROI; orientation classification enabled (cls = True) (Section 3.4 and Section 4.2)
Decision parameters	Encroachment threshold *τ* = 0.20 (default; Section 4.9); temporal counter *N* = 3 (default; Section 3.1.2 or Section 4.8)
Hardware	NVIDIA GeForce RTX 4090 GPU (Section 4.1)

**Table 2:** Comparison of license plate recognition performance among different OCR methods.

OCR Methods	CA (%)	PA (%)	FPS	Average Inference Time (ms)
LPRNet	88.98	40.00	117.96	8.48
EasyOCR	86.72	30.00	8.44	118.43
PaddleOCR	96.61	76.00	22.26	44.93

**Table 3:** Performance comparison of different OBB detection models.

Model	mAP50 (%)	mAP50-95 (%)	Precision (%)	Recall (%)	Val Box Loss	State
YOLOv8n-OBB	94.6	80.8	93.7	86.2	0.591	Early stopping (Ep 54)
YOLOv11n-OBB	95.8	84.8	89.8	91.0	0.523	Done (100 eps)
YOLOv5n-OBB	96.5	86.6	93.0	91.1	0.482	Done (100 eps)

**Table 4:** Cross-validation experimental design.

Experiment Number	Train Set	Test Set	Purpose
Exp 1	CCPD	CCPD	[Baseline A] Evaluate the theoretical performance upper bound of the model in standard and normalized scenarios.
Exp 2	Drone	Drone	[Baseline B] Evaluate the model’s learning capability in complex scenarios with top-down views and large-angle rotations.
Exp 3	CCPD	Drone	[Cross-validation] Test whether the model trained only on street-view frontal features can handle extreme rotation perspectives.
Exp 4	Drone	CCPD	[Cross-validation] Test whether the model trained with multi-dimensional features can be downward compatible with standard scenarios.

**Table 5:** Summary of detection performance under different data combination perspectives.

Experiment	mAP50	mAP50-95	Conclusion Summary
Exp 1	0.986	0.926	Ultra-high precision
Exp 2	0.965	0.869	Excellent performance
Exp 3	0.644	0.495	Limited generalization
Exp 4	0.931	0.795	Strong generalization

**Table 6:** Semantic segmentation performance on non-motorized vehicle lanes.

Metric	Value
IoU	39.63%
Dice	55.30%
Precision	46.13%
Recall	72.35%
F1-score	55.30%
FPS	22.1

**Table 7:** Scenario-wise decision performance on the Drone 100-sample evaluation set.

Scenario	*n*	Pos	Neg	Accuracy (%)	Precision (%)	Recall (%)	F1 (%)	FPR (%)
Straight road	19	19	0	94.74	100.00	94.74	97.30	0.00
Diagonal/oblique view	27	27	0	100.00	100.00	100.00	100.00	0.00
Borderline near lane edge	20	20	0	75.00	100.00	75.00	85.71	0.00
Brief boundary interaction	13	0	13	92.31	N/A ^1^	N/A	N/A	7.69
Occlusion/crowded	21	0	21	95.24	N/A	N/A	N/A	4.76
Overall	100	66	34	92.00	96.77	90.91	93.75	5.88

^1^ Some scenario subsets are defined by visual condition or boundary interaction patterns rather than by the presence of positive violations. Therefore, certain subsets may contain no positive samples (Pos = 0). For these subsets, precision, recall, and F1-score are reported as N/A because positive-class evaluation is undefined.

**Table 8:** Performance comparison of different encroachment detection methods.

Metric	Baseline (HBB + Center Point)	OBB Reference (Area + Temporal Sequence)
Accuracy	67.00%	92.00%
Precision	80.00%	96.77%
Recall	66.67%	90.91%
F1-score	72.73%	93.75%
False Positive Rate (FPR)	32.35%	5.88%
Balanced Accuracy	67.16%	92.51%
MCC	32.68%	82.98%

**Table 9:** System component ablation results and end-to-end runtime of the complete system.

Group	License Plate Recognition Rate (%)	Violation Accuracy (%)	Violation Recall (%)	F1 (%)	FPS	p50 Latency (ms)	p95 Latency (ms)
G1	28.33	60.00	52.94	65.22	40.2	-	-
G2	43.33	75.00	65.88	78.87	34.0	-	-
G3	57.50	85.83	85.88	89.57	20.7	-	-
G4 *	69.17	80.00	76.47	84.42	16.3	61.44	75.32

* p50/p95 latency was measured only for the complete deployed system (G4) on the target hardware.

**Table 10:** Stability comparison of HBB-based decision strategies (temporal debounce).

Test Group	FPR (%)	Stability (%)	Response Delay (Frames)
Single-frame HBB	37.1	56.1	0.2
Temporal HBB (*N* = 3)	7.6	80.0	1.6

## Data Availability

The public datasets used in this study, including CCPD and BDD100K, are available from their original sources. The self-collected Drone dataset contains identifiable vehicle and license plate imagery and is therefore subject to privacy and compliance constraints under applicable institutional and local data governance policies. To support reproducibility while protecting personal information, the authors can provide an anonymized version of the evaluation data (e.g., plate number masking and/or obfuscated crops), derived annotations (vehicle HBBs, plate OBBs, and lane masks), and the evaluation protocol upon reasonable request. In addition, the training/inference code, configuration files, and evaluation scripts will be released in a public repository upon acceptance. If raw frames cannot be publicly redistributed, we will release a reproducible obfuscated subset or equivalent evaluation package sufficient to reproduce the reported metrics.
